# Supplementary material for: Weak Effect of Gypsy Retrotransposon Bursts on Sonneratia alba Salt Stress Gene Expression
Source: Front Plant Sci. 2022 Jan 17;12:830079. doi: 10.3389/fpls.2021.830079 (PMC8801733; doi:10.3389/fpls.2021.830079)
Supplement: Supplementary file 1 [file Image_1.PDF]

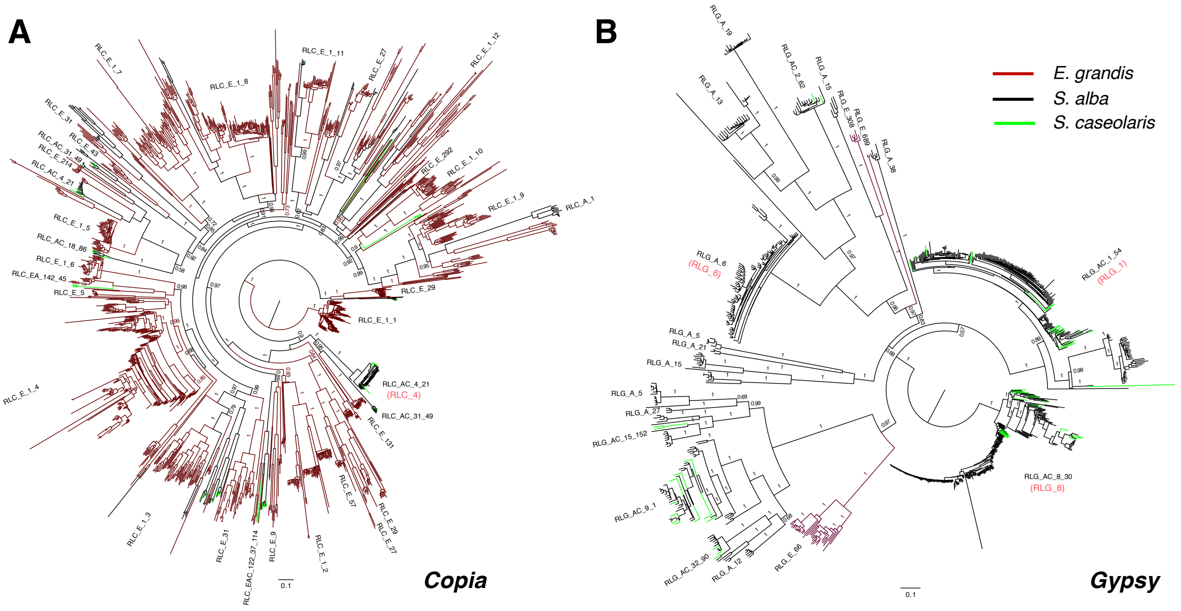

Supplementary Figure 1 Maximum likelihood (ML) phylogenetic trees of *Copia* and *Gypsy* LTR retrotransposon families. Sequences of intact (A) *Copia* and (B) *Gypsy* copies identified in *S. alba* and *S. caseolaris* were used for this analysis. Supporting values of each branch were calculated by resampling estimated log-likelihoods (RELL)-like local support values.
