# Supplementary material for: Weak Effect of Gypsy Retrotransposon Bursts on Sonneratia alba Salt Stress Gene Expression
Source: Front Plant Sci. 2022 Jan 17;12:830079. doi: 10.3389/fpls.2021.830079 (PMC8801733; doi:10.3389/fpls.2021.830079)
Supplement: Supplementary file 2 [file Image_2.PDF]

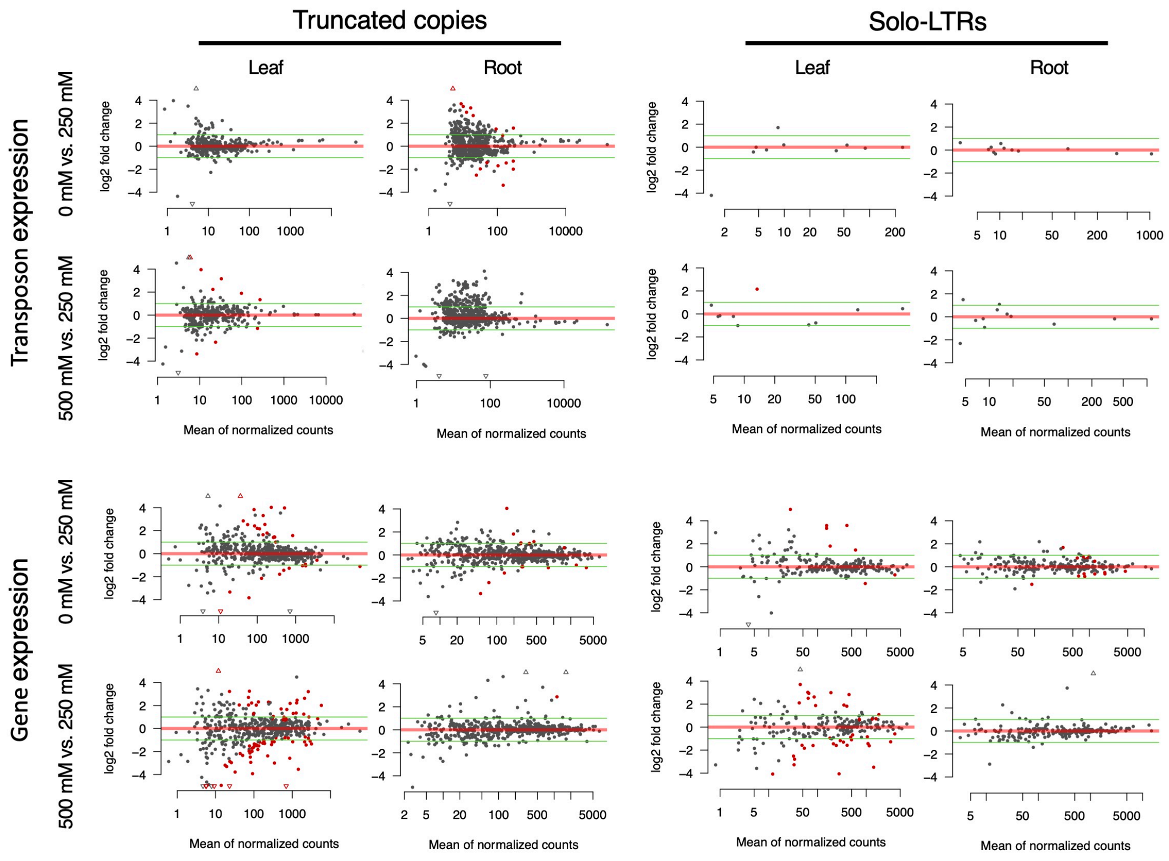

Supplementary Figure 2 Expression changes of transposons and their nearest genes across salt treatments in leaves and roots. Both the truncated LTR retrotransposons and solo-LTRs were investigated. TEs or genes with significant mis-expression (Benjamini-Hochberg FDR < 0.05) between treatments are indicated in red. Green lines indicate two-fold expression changes.
