# Supplementary material for: Weak Effect of Gypsy Retrotransposon Bursts on Sonneratia alba Salt Stress Gene Expression
Source: Front Plant Sci. 2022 Jan 17;12:830079. doi: 10.3389/fpls.2021.830079 (PMC8801733; doi:10.3389/fpls.2021.830079)
Supplement: Supplementary file 3 [file Image_3.PDF]

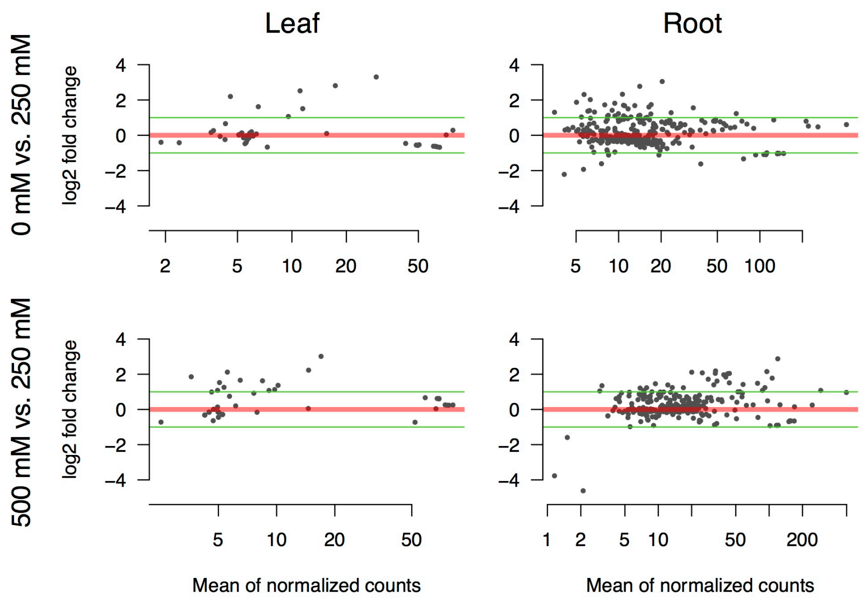

Supplementary Figure 3 Expression level fold change of intact RLG_1 and RLG_8 retrotransposons across salt treatments. Upper panels: 0 mM vs 250 mM NaCl, lower panels: 250 mM vs 500 mM NaCl. Transposon elements with significant mis-expression (Benjamini-Hochberg FDR < 0.05) between treatments are indicated in red.
