# Supplementary material for: Weak Effect of Gypsy Retrotransposon Bursts on Sonneratia alba Salt Stress Gene Expression
Source: Front Plant Sci. 2022 Jan 17;12:830079. doi: 10.3389/fpls.2021.830079 (PMC8801733; doi:10.3389/fpls.2021.830079)
Supplement: Supplementary file 4 [file Presentation_1.PDF]

Supplementary Note

The impact of TE expansion on gene expression divergence between *S. alba* and *S. caseolaris*

We examined the impact of the top two abundant TE families (RLG_1 and RLG_8) on expression divergence between *S. alba* and *S. caseolaris*. RNA-seq was conducted using leaves of *S. alba* and *S. caseolairs* collected from plants growing in Qinlan Harbour, Hainan, China (Wang et al. unpublished data). Expression level was calculated as described in the Materials and Methods. There are 712 genes associated with the RLG_1 or RLG_8 elements (< 3,000 bp upstream or downstream) in *S. alba*, and 165 genes in *S. caseolaris*. All the intact and truncated elements and solo-LTRs were considered. For each gene associated with RLG_1 or RLG_8 in each species, we first identified their orthologous genes in the other species using BLASTn and a cutoff of $e \leq 10$, and then checked whether TE from the same family is present within 10,000 bp upstream or downstream of the orthologous gene. By doing so, we were able to classify all the RLG_1- or RLG_8-associated genes into three groups (*S. alba*/*S. caseolaris*: +/-, -/+ and +/+). Based on the transcriptome data under normal condition, we calculated the between-species expression divergence for each gene group as 1-ρ, where ρ is the Pearson correlation coefficient of gene expression levels between *S. alba* and *S. caseolaris*. Finally, we compared the observation with the distribution of expression divergence genome wide, which was calculated by randomly sampling a subset of genes from the total number of expressed genes in *S. alba* for 1000 times. The number of genes sampled for RLG_1 or RLG_8 is the total number of genes associated with this family in the two species.

Only the set of genes associated with RLG_1 in *S. alba* but not in *S. caseolaris* showed significantly higher expression divergence than the expectation genome wide (Permutation test, $P < 0.02$, Figure SN1). Considering a substantial fraction of RLG_1 elements exhibited high CHH methylation in genic regions (Figure 4A), we think the observed increase of expression divergence is more likely because RLG_1 in *S. alba* carried repressive epigenetic markers rather than they created new regulatory elements. Consistent with the view, no RLG_1 copy was found to be candidate of TE co-option although RLG_1 is the most abundant TE family in *S. alba.*

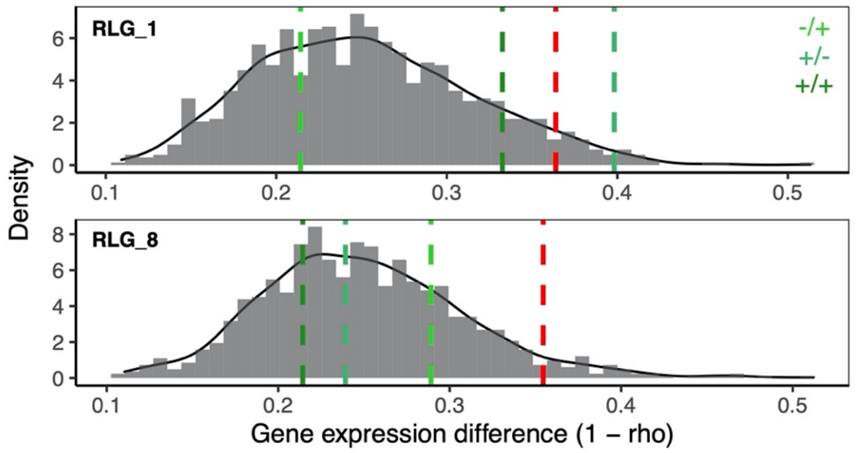

Figure SN1. Expression divergence of the RLG_1- and RLG_8-associated genes between *S. alba* and *S. caseolaris*. The histogram represents the empirical distribution of gene expression difference (1-rho) between *S. alba* and *S. caseolaris* after 1,000 bootstrapping. The dotted line in red represents the expression divergence at the significance level of 0.05. The dotted lines in different green colors represent the observed expression divergence for genes associated with RLG_1 or RLG_8 in *S. caseolaris* but not in *S. alba* (-/+, neon green); +/-, genes associated with RLG_1 or RLG_8 in *S. alba* but not in *S. caseolaris* (+/+, forest green) and genes associated with RLG_1 or RLG_8 in both species (+/+, dark green)."
