# Supplementary material for: Weak Effect of Gypsy Retrotransposon Bursts on Sonneratia alba Salt Stress Gene Expression
Source: Front Plant Sci. 2022 Jan 17;12:830079. doi: 10.3389/fpls.2021.830079 (PMC8801733; doi:10.3389/fpls.2021.830079)
Supplement: Supplementary file 7 [file Table_3.DOCX]

**Supplementary Table 3**. The number of TE-adjacent genes in *Sonneratia alba* that were significantly mis-regulated by salt treatment. Only those from the top four most abundant LTR retrotransposon families were counted.

|  |  | Leaf 0 vs. 250 mM | Root 0 vs. 250 mM | Leaf 500 vs. 250 mM | Root 500 vs. 250 mM |
| --- | --- | --- | --- | --- | --- |
| RLG_8 | Differentially expressed | 2 | 3 | 18 | 0 |
|  | Total expressed | 108 | 99 | 106 | 126 |
| RLG_1 | Differentially expressed | 11 | 2 | 14 | 1 |
|  | Total expressed | 165 | 159 | 155 | 193 |
| RLG_6 | Differentially expressed | 8 | 5 | 25 | 0 |
|  | Total expressed | 162 | 145 | 162 | 203 |
| RLC_4 | Differentially expressed | 4 | 1 | 8 | 0 |
|  | Total expressed | 46 | 41 | 44 | 57 |
| Whole transcriptome | Differentially expressed | 1,212 | 390 | 2,835 | 59 |
|  | Total expressed | 26,326 | 27,243 | 26,326 | 27,243 |
