# Supplementary material for: Weak Effect of Gypsy Retrotransposon Bursts on Sonneratia alba Salt Stress Gene Expression
Source: Front Plant Sci. 2022 Jan 17;12:830079. doi: 10.3389/fpls.2021.830079 (PMC8801733; doi:10.3389/fpls.2021.830079)
Supplement: Supplementary file 11 [file Table_7.DOCX]

**Supplementary Table 7**. Pairs of LTR retrotransposons and genes with potential miRNA competition in *S. alba.* Pearson correlation was calculated for expression levels of each pair across 0mM, 250 mM and 500mM salt treatments in roots and leaves, respectively. *cor*, Pearson correlation coefficient; P, P value.

| LTR elements | Shared miRNA | miRNA sequence | Gene | Leaf | |  | Root | |
| --- | --- | --- | --- | --- | --- | --- | --- | --- |
|  |  |  |  | *cor* | P |  | *cor* | P |
| SalLTR13_scaffold1_7261969_7268972 | miR3937 | acaggcgguggaucaaauaugaau | evm.model.scaffold11.642 | -0.14 | 0.79 |  | 0.60 | 0.21 |
| SalLTR6_scaffold50_292888_294854 | miR5622 | auuagcuguugggacuuaaaagcc | evm.model.scaffold39.173 | -0.74 | 0.09 |  | 0.78 | 0.07 |
|  |  |  | evm.model.scaffold12.225 | -0.44 | 0.39 |  | -0.02 | 0.97 |
| SalLTR6_scaffold50_831587_837043 | miR4239 | auuguuauuuuguuggaccggccu | evm.model.scaffold14.309 | -0.37 | 0.47 |  | 0.06 | 0.91 |
| SalLTR6_scaffold50_881694_887933 |  |  | evm.model.scaffold3.1127 | -0.61 | 0.20 |  | -0.07 | 0.90 |
| SalLTR6_scaffold50_878576_885125 |  |  | evm.model.scaffold22.100 | -0.31 | 0.55 |  | -0.08 | 0.88 |
| SalLTR1_scaffold5_755227_760280 | miR8051 | uauuucuuggacucggcuuguaac | evm.model.scaffold54.63 | -0.12 | 0.82 |  | -0.02 | 0.97 |
